# Peer-Mediated Digital Awareness Among Adolescents: Insights from a CAWI-Based Assessment at the European Researchers’ Night

**DOI:** 10.3390/bs16030469

**Published:** 2026-03-21

**Authors:** Daniele Giansanti, Lorenzo Desideri, Antonia Pirrera, Regina Gregori Grgič

**Affiliations:** 1 National Center IATIS, Istituto Superiore di Sanità-Roma, 00161 Roma, Italy; antonia.pirrera@iss.it; 2 Digital Psychology Lab, Sigmund Freud University, 20143 Milano, Italy; l.desideri@milano-sfu.it (L.D.); r.gregori@milano-sfu.it (R.G.G.)

**Keywords:** smartphone, addiction, adolescents, mobile phone, computer-aided web interviewing, CAWI

## Abstract

Adolescents increasingly engage with digital technologies, yet understanding patterns of smartphone use and fostering reflective awareness remain challenging. Traditional assessments in clinical or school settings may limit participation and self-reflection. This study evaluated the feasibility and impact of a Computer-Assisted Web Interviewing (CAWI) approach to monitor smartphone use, provide immediate individualized feedback, and support peer-mediated dissemination in a public science engagement context. Across three editions of the European Researchers’ Night in Rome (2023–2025), 807 adolescents aged 10–19 completed the SAS-SV questionnaire via on-site tablets or personal devices using QR codes. Smartphone use was categorized into Low Involvement, At-Risk, or Problematic. Participants were encouraged to share the survey link with peers, enabling snowball-mediated recruitment. Participant acceptance was assessed through the Net Promoter Score (NPS). Snowball participation accounted for the majority of responses, highlighting the effectiveness of peer-mediated diffusion. SAS-SV categorization indicated 46% Low Involvement, 39% At-Risk, and 15% Problematic use, with minimal gender differences. NPS values ranged from +69 to +79, with snowball participants reporting slightly higher satisfaction than on-site attendees. These results underscore high engagement, perceived value, and the role of peer networks in promoting reflective digital behavior. Integrating CAWI assessment, immediate feedback, and peer-mediated diffusion created a socially situated environment supporting self-reflection and voluntary dissemination. Peer networks extended both the temporal and social reach of the initiative beyond the public event, demonstrating a scalable and non-stigmatizing model. CAWI-based monitoring combined with feedback and peer-driven diffusion is feasible and effective for adolescent digital wellbeing interventions. This approach fosters reflective digital citizenship, supports self-awareness, and leverages social networks to enhance the reach and impact of youth-centered health promotion initiatives.

## 1. Introduction

### 1.1. Adolescent Smartphone Engagement: Theory and Evidence

The evolution of the smartphone, from its early development and global diffusion (Agar, 2013; Garcia-Swartz & Campbell-Kelly, 2022) to the revolutionary impact of the iPhone and the reshaping of the mobile industry landscape (Merchant, 2017; McCullough, 2018; Vogelstein, 2013), has progressively transformed mobile devices into pervasive, intelligent interfaces that influence social behavior, cognition, and daily life (Saylor, 2012; Isaacson, 2014; Montag & Diefenbach, 2018). Among adolescents, daily usage often exceeds three to five hours, with many teenagers spending even more time engaged with apps and social media (Giansanti & Maccioni, 2021a, 2021b). This pervasive use raises concerns about potential negative outcomes, both physically and psychologically (The Lancet Child & Adolescent Health, 2018).

Physically, prolonged smartphone engagement is associated with musculoskeletal issues, including neck and shoulder strain—commonly referred to as “text neck” (Toh et al., 2017)—as well as eye strain (Neupane et al., 2017) and postural fatigue (Lee et al., 2015; Hansraj, 2014; Choi et al., 2016; Cuéllar & Lanman, 2017). Psychologically, excessive use can correlate with increased feelings of loneliness (Peper & Harvey, 2018), anxiety (De Pasquale et al., 2015), and depressive symptoms (Elhai et al., 2017), alongside patterns of compulsive engagement resembling behavioral addiction (Kwon et al., 2013). To identify at-risk individuals, validated instruments such as the Smartphone Addiction Scale (SAS) (Lopez-Fernandez, 2017) and its short versions (SAS-SV) (De Pasquale et al., 2017) are widely applied in adolescent populations, allowing researchers to measure both the intensity and the functional impact of smartphone use.

The term “smartphone addiction” is commonly used in research, though some scholars prefer “problematic smartphone use” (PSU) to indicate functional difficulties without implying a clinical disorder (Panova & Carbonell, 2018; Billieux et al., 2015). PSU may manifest through frequent checking, prolonged usage, and difficulty disengaging, highlighting the need for accurate assessment tools and intervention strategies. This distinction is central to our study, which focuses on adolescents in public science engagement contexts and seeks to understand behavioral patterns without pathologizing normative use.

The Interaction of Person–Affect–Cognition–Execution (I-PACE) model offers a framework for understanding these behaviors (Brand et al., 2019; Mehmood et al., 2021). It describes how individual predispositions (e.g., personality traits, impulsivity), affective states (e.g., stress, boredom), cognitive biases (e.g., attention to rewarding stimuli), and executive functioning capacities (e.g., self-regulation) interact to influence smartphone use. Empirical applications of the I-PACE model demonstrate its relevance across age groups and cultural contexts (Mehmood et al., 2021), providing a theoretical justification for the selection of measurement instruments such as SAS/SAS-SV and for interpreting patterns observed among adolescents during engagement activities.

Neurobiological and psychosocial factors further shape adolescent smartphone behavior. Engagement activates reward pathways, including dopaminergic circuits, reinforcing habitual use (Montag et al., 2021; Horvath et al., 2020). Social drivers, such as the desire for connectedness, Fear of Missing Out (FoMO), and coping strategies, modulate engagement patterns (Przybylski et al., 2013; Hamamura et al., 2023).

Moreover, the environment, whether supportive or high-pressure, can influence the intensity and nature of smartphone interactions (Hamamura et al., 2023). Understanding these factors is critical for interpreting empirical data collected through tools like CAWI surveys, where adolescents can self-report behaviors and engage in reflective feedback processes, facilitating both research insights and educational outcomes.

### 1.2. Integrating Monitoring, Education, and Public Engagement: The ISS Strategy for Adolescent Smartphone Awareness

Smartphone overuse can have negative physical, psychological, and social consequences, making the promotion of awareness and a mindful smartphone culture particularly important. A narrative review of recent literature (Giansanti, 2025) highlights the growing concern around adolescent smartphone engagement and the need for structured approaches to monitor usage and potential risks.

Validated instruments, such as the Smartphone Addiction Scale (SAS) and its Short Version (SAS-SV) (Kwon et al., 2013; De Pasquale et al., 2017), provide a necessary framework for assessing smartphone use among adolescents. Structured monitoring using these tools allows researchers and educators to identify patterns of engagement, potential risk behaviors, and areas where targeted educational interventions can support knowledge, digital literacy, and shared responsibility within communities.

The Italian National Institute of Health (Istituto Superiore di Sanità, ISS) provides a national model for integrating research, education, and public engagement. Over the past decade, ISS has developed a multifaceted approach including scientific publications, educational campaigns, digital tools, and public engagement activities. ISTISAN reports offer methodological frameworks, guidance for data collection, and validated instruments for assessing digital dependence (Giansanti, 2019; Giansanti & Grigioni, 2018).

Youth engagement has been a particularly innovative component. Programs such as school-to-work transition initiatives (Alternanza Scuola-Lavoro, ASLP) allow students to participate directly in research, data collection, and communication activities. These initiatives foster critical digital literacy while acting as multipliers of preventive messages (Salinetti et al., 2018; Giansanti et al., 2019). Surveys administered via CAWI (Computer-Assisted Web Interviewing) provide adolescents with a simple, anonymous, and scalable tool for assessing smartphone use. Inclusion of instruments such as the SAS and SAS-SV enables both accurate measurement and immediate participant feedback (Istituto Superiore di Sanità, n.d.-a).

ISS’s public engagement extends to large-scale events like the European Researchers’ Night, where interactive workshops and exhibits—e.g., “Lo smartphone, un amico ma non troppo”—encourage adolescents and families to reflect on digital well-being in an engaging, playful context (Istituto Superiore di Sanità, n.d.-b). These activities integrate assessment with cultural sensitization, supporting awareness while generating actionable data (Istituto Superiore di Sanità, n.d.-a, 2020).

Taken together, ISS initiatives exemplify a three-pronged approach to promoting healthy smartphone use among adolescents:

- Monitoring digital behavior: Validated instruments like SAS and SAS-SV, administered via CAWI, allow rapid, scalable, and anonymous assessment of usage patterns and risk.
- Promoting a culture of awareness: Educational campaigns, school-based programs, and interactive workshops foster critical digital literacy and encourage responsible, mindful use.
- Immediate feedback and empowerment: CAWI-based surveys provide participants with insights into their own habits, creating a self-awareness loop and promoting informed behavior.

By integrating monitoring, cultural sensitization, and immediate feedback, this framework aligns scientific research with educational and civic engagement goals, providing a reproducible model for local and national digital health strategies (Giansanti, 2019; Giansanti & Grigioni, 2018; Salinetti et al., 2018; Giansanti et al., 2019; Istituto Superiore di Sanità, n.d.-a, 2020).

### 1.3. Aim of the Study

The aim of this initiative was to track smartphone usage among adolescents in real-world educational and science engagement contexts, while simultaneously raising awareness of healthy digital habits. Specifically, the project sought to:

1. Assess smartphone use patterns during large-scale public science events, leveraging a validated tool (SAS-SV) to identify trends and potential risk factors.
2. Engage adolescents in reflection and learning, by providing immediate feedback on their responses to the questionnaire and fostering awareness of responsible smartphone use.
3. Test the feasibility of dual data collection methods—on-site administration via CAWI terminals and distributed participation through QR codes—allowing for both immediate participation and extended reach beyond the event.

This work was conducted during three consecutive editions of the European Researchers’ Night, hosted by the ISS, where adolescents naturally gather to explore science, technology, and health topics. By focusing explicitly on adolescents, the initiative integrated research, education, and community engagement, ensuring that data collection remained both scientifically robust and socially meaningful.

*Rationale for terminology:* Although the term “youth” is commonly used in the literature as a broader category, the present study focuses specifically on adolescents (roughly ages 10–19), corresponding to middle and high school populations. This clarification ensures conceptual and analytical consistency throughout the manuscript.

## 2. Materials and Methods

### 2.1. Setting and Participants

The present study was conducted within the framework of the European Researchers’ Night, a Europe-wide public science outreach initiative supported by the European Commission under the Marie Skłodowska-Curie Actions (MSCA) and Citizens scheme (Horizon Europe).This initiative promotes public engagement with science across multiple European cities through approved educational and interactive activities. The Rome/Frascati edition was locally coordinated by Frascati Scienza and took place at the “NET Village” in the Città dell’Altra Economia (Testaccio, Rome), providing a dedicated setting for science communication and public participation. Data were collected during the last Friday and Saturday of September 2023, September 2024, and September 2025, forming a continuous, three-year observational design. The inclusion of all three editions allows the study to capture temporal trends, natural variations in participation, and the progressive impact of public engagement efforts, providing a more robust understanding of adolescent digital awareness than a single-year snapshot.

The target population initially included adolescents and young adults (approximately 10–25 years old) attending the events. For the purpose of this study, analyses were restricted to adolescents aged 10–19 years to ensure sample homogeneity, cultural and educational comparability, and meaningful interpretation of engagement patterns across age groups. Participation in the survey was voluntary, fully anonymous, and non-interventional, with no personal identifiers recorded. Respondents accessed the questionnaire via a QR code linked to a secure online form, enabling self-administration on site during the event or afterwards, when participants shared the link within their networks (snowball diffusion). The questionnaire was administered through Microsoft Forms (Microsoft Corporation, Redmond, WA, USA), a web-based platform within the Microsoft 365 institutional suite. Microsoft Forms is continuously updated by the provider, with no fixed version number, and the latest updates were available at the time of data collection. Microsoft Forms was selected, as it is widely used for academic data collection and has been extensively applied in survey-based research, particularly in the public health domain (Shiju et al., 2023). Demographic information collected included age, gender, school level, and educational context. Participants were primarily enrolled in middle and high schools, reflecting the typical adolescent population attending public science engagement events in the Rome metropolitan area and surrounding towns, ensuring a broad, culturally coherent sample. The inclusion of this geographic and educational information supports interpretation of observed engagement patterns and ensures relevance for the adolescent community studied.

Data collection was non-interventional, observational, fully anonymous, and conducted within a publicly approved, non-clinical, educational science outreach project funded by the European Union. Under applicable national and European ethical guidelines for anonymous, non-interventional educational activities, formal Institutional Review Board (IRB) approval was not required. The initiative itself underwent public and institutional approval for science outreach, providing full legitimacy and oversight of the educational activities and survey administration. Nonetheless, all procedures adhered to the principles of the Declaration of Helsinki and relevant European Union data-protection regulations (GDPR). At the start of the CAWI survey, participants were informed about the initiative, the purpose of the survey, the voluntary nature of participation, and their option not to take part. This approach ensured transparency, respected participants’ autonomy, and safeguarded anonymity.

This triennial project design was intentionally implemented to observe a continuous educational and engagement initiative over time. Conducting the study across three consecutive editions allowed for monitoring trends, assessing the sustainability of peer-driven awareness strategies, and evaluating the cumulative effects of repeated interventions. Such a longitudinal perspective strengthens the insights gained on adolescent engagement with digital tools, while remaining cautious about generalization beyond the observed sample.

### 2.2. Procedure

On-site, participants were invited to complete the Smartphone Addiction Scale—Short Version (SAS-SV) through a CAWI interface accessible from dedicated tablets located at the ISS booth. Simultaneously, the survey was accessible by scanning a QR code displayed during the event. This allowed participants to complete the questionnaire later, on their personal devices, and to easily share the link within their social networks. This dual on-site/remote access model facilitated both immediate engagement and subsequent diffusion, enabling a natural snowball effect: participants who first encountered the survey during the event could later share the QR code with peers, gradually expanding participation beyond the physical context of the European Researchers’ Night.

Data collection was conducted using a CAWI approach through Microsoft Forms, a platform integrated into the Microsoft 365 suite adopted institutionally by the ISS. This environment is certified for internal research and educational activities, aligns with ISS data protection and cybersecurity standards, and operates under the European General Data Protection Regulation (GDPR). Importantly, the system does not record IP addresses or geolocation data, ensuring full anonymity of participants. However, Microsoft Forms automatically records the date and time of completion, which was crucial for analyzing participation trends during the events and in the days that followed due to peer sharing.

The survey included a brief demographic section (age, gender, and school level) to support interpretation of engagement patterns within the Rome metropolitan area adolescent community, as well as a self-orientation section that allowed participants to understand their responses across the three SAS-SV interpretative ranges: low involvement, at-risk use, and problematic use. This provided an opportunity for self-reflection on smartphone use without generating identifiable or clinically diagnostic data.

In addition, a single-item Net Promoter Score (NPS) question was embedded directly in the CAWI survey (Qualtrics, 2025; SurveyMonkey, 2025; CustomerGauge, 2025).

The NPS asks participants how likely they would be to recommend the activity to a peer on a scale from 0 (not at all likely) to 10 (extremely likely). In general, the NPS is widely used in experience evaluation to measure satisfaction, perceived value, or engagement. In this study, the NPS served two purposes: (1) to assess the perceived relevance and resonance of the initiative among adolescents, and (2) to estimate the potential for peer-driven diffusion, which underlies the snowball effect observed when participants shared the survey link within their networks. The NPS is not a clinical or diagnostic tool, but a simple, reliable indicator of participant experience and social engagement dynamics.

Upon completion of the SAS-SV, participants received interpretative guidance corresponding to the three reference ranges (low involvement, at-risk use, problematic use), supporting individual reflection on smartphone habits without displaying a numerical score or storing identifiable data.

Data from both on-site and remote submissions were recorded in a secure, anonymized institutional repository. While individual responses could not be traced back to participants, the database enabled descriptive and comparative analyses across age groups, event editions, and participation modalities. Participation was voluntary, and informed consent was implied through survey completion. This approach ensured a balance between ethical protection, operational feasibility, and the dual objectives of awareness promotion and population-level monitoring.

This design underscores the dual role of smartphones in the study: they are both the object of measurement and the medium through which participants engage in self-assessment and awareness. By completing the questionnaire on their own devices, participants were able to review and reflect on their responses.

### 2.3. Data Analysis and Snowball Effect

Data were analyzed using STATA version 16 (StataCorp, College Station, TX, USA) to evaluate overall engagement patterns, SAS-SV response distributions, and participation metrics across the three consecutive editions of the European Researchers’ Night (2023–2025). Analyses focused on aggregated data only, since no personal identifiers, IP addresses, or geolocation information were collected.

The date and time stamps automatically recorded by Microsoft Forms allowed differentiation between on-site participation (during the event) and post-event participation resulting from the snowball effect, in which participants shared the QR code with peers in the hours and days following each edition. To quantify this effect, the snowball effect was calculated as:

The Snowball Effect, calculated as

(1) $$Snowball\;effect=\frac{N_{snowball}}{N_{on\text{-}site}}$$

quantifies how many additional participants were recruited via remote sharing for each on-site attendee.

Where *N_snowball_* is the number of participants who completed the survey remotely via peer sharing, and *N_on-site_* is the number of participants who completed the survey during the event.

This measure describes the extent to which peer-mediated sharing contributed to the total number of participants.

The repeated cross-sectional design allowed comparisons across age, gender, school level, and participation modality (on-site vs. snowball). Chi-squared tests were performed to examine distributions across SAS-SV interpretative ranges (low involvement, at-risk use, problematic use) and to compare participation patterns across editions.

## 3. Results

The present results focus exclusively on adolescent participants (10–19 years old) recruited across three consecutive editions of the European Researchers’ Night (2023–2025) in the Rome metropolitan area and surrounding towns. Data are organized in a structured, hierarchical manner to provide a clear understanding of both participation dynamics and smartphone engagement patterns:

1. Participant Distribution and Snowball Effect (Section 3.1):

Participant counts are presented by year, mode of participation (on-site vs. snowball), age, gender, and school level (middle vs. high school). The Snowball Effect quantifies peer-mediated participation beyond the on-site events, highlighting both its magnitude and temporal decay over the weeks following each edition.

2. SAS-SV Risk Classification (Section 3.2):

Adolescents’ responses on the Smartphone Addiction Scale—Short Version (SAS-SV) are classified into three gender-specific risk categories: low involvement, at-risk use, and problematic use. Results are presented by year and gender, and chi-square tests evaluate stability of risk distributions across editions as well as deviations from a uniform distribution, confirming the predominance of low involvement and at-risk use.

3. Net Promoter Score (NPS) and Relationship with SAS-SV (Section 3.3):

Participants’ perceived value of the initiative was assessed using the NPS single-item question (0–10 scale). Responses are classified into Promoters, Passives, and Detractors, with percentages reported for each year and by mode of participation. The association between SAS-SV categories and NPS classifications is explored using a Chi-square test of independence, providing a preliminary view of how smartphone engagement patterns relate to participants’ willingness to recommend the initiative.

This structure ensures that results are presented clearly and logically, moving from descriptive participation data to behavioral measures (SAS-SV) and then to evaluative feedback (NPS), while highlighting key dynamics such as peer-mediated diffusion, gender-specific engagement, and risk perception.

### 3.1. Partecipant Distribution and Snowball Effect

The study included a total of 807 adolescents aged 10–19 years who participated across the three editions of the European Researchers’ Night (2023–2025). All participants were recruited in the Rome metropolitan area and surrounding towns. The sample was composed mainly of middle school students, with high school students representing a smaller proportion. Gender distribution was balanced, with females slightly more represented than males.

Participation took place either on-site during the event or remotely through QR-code sharing in the days afterward. The distribution of participants by year, participation mode, school level, and gender is summarized in Table 1.

In 2023, there were 48 on-site participants and 139 remote participants, for a total of 187 adolescents. In 2024, on-site participants increased to 59 and remote participants to 201, totaling 260. By 2025, on-site participants reached 62, with 298 joining via the snowball effect, yielding a total of 360 adolescents. The mean age of participants remained relatively stable across the three years, ranging from 16.5 ± 1.9 in 2023 to 16.9 ± 2.1 in 2025. Middle school students consistently represented the majority of the sample (74–78%), while high school students accounted for 22–26%. Female participants ranged from 52–54% across editions, with males representing 46–48%.

The Snowball Effect, calculated as the number of additional participants recruited for each on-site attendee, increased from 2.90 in 2023 to 4.80 in 2025. This indicates a growing contribution of peer-mediated participation over time, emphasizing the social diffusion component of the study. Each on-site adolescent recruited on average 2.9 additional participants in 2023, 3.4 in 2024, and 4.8 in 2025.

To assess whether the distribution of participation mode differed across editions, a chi-square test of independence was performed. Results indicated a statistically significant difference across years (χ^2^ test, df = 2, *p* < 0.05) with a small effect size (Cramer’s V = 0.086), indicating a slight but measurable increase in remote participation relative to on-site attendance over the three editions.

The snowball effect demonstrates both the magnitude and persistence of peer-mediated participation following each European Researchers’ Night edition. Across the three years, responses collected via the snowball mechanism continued to arrive for up to three weeks after the on-site events, reflecting a natural decay process in participation over time. In 2023, of the 139 snowball responses, 110 (79%) were received within the first 10 days, with the remaining 29 (21%) arriving during days 11–21, completing the three-week period. In 2024, 201 snowball responses included 162 (81%) collected in the first 10 days and 39 (19%) over days 11–21. Similarly, in 2025, 298 snowball responses comprised 240 (81%) early responses and 58 (19%) during days 11–21. This pattern shows a rapid initial engagement followed by a gradual decline in responses, highlighting the temporal dynamics of peer-mediated survey diffusion among adolescents.

### 3.2. SAS-SV Scores

The SAS-SV responses for adolescent participants were classified into three interpretative ranges based on gender-specific thresholds established by (Kwon et al., 2013): for males, low involvement (≤21), at-risk use (22–31), and problematic use (>31); for females, low involvement (≤22), at-risk use (23–33), and problematic use (>33). These thresholds allow for a gender-sensitive evaluation of smartphone engagement and potential addiction risk.

Table 2 presents the distribution of adolescents across these risk categories by year and gender, combining both on-site and snowball responses.

Across the three editions, the majority of adolescents fell within the low involvement or at-risk categories, with problematic use representing a smaller proportion that shows a slight increase over time. For instance, problematic use among female participants increased from 13% in 2023 to 15% in 2025, while for male participants it ranged from 12% to 17% over the same period.

A Chi-square test of independence by gender indicated no significant differences in SAS-SV risk distributions between males and females for 2023 (df = 2, *p* > 0.05), 2024 (df = 2, *p* > 0.05), and 2025 (df = 2, *p* > 0.05).

Additionally, a Chi-square test of independence across editions (df = 4, *p* > 0.05) was performed to evaluate whether the distribution of SAS-SV risk categories differed significantly between years. The results suggest that patterns of smartphone engagement among adolescents remained stable over the three editions, despite slight variations in the proportion of problematic users.

Finally, a Chi-square goodness-of-fit test for each year confirmed that the observed distributions were significantly non-uniform (2023: df = 2, *p* < 0.001; 2024: df = 2, *p* < 0.001; 2025: df = 2, *p* < 0.001), reflecting the predominance of low involvement and at-risk use compared to problematic use.

This combined analysis provides a robust, population-level overview of adolescent smartphone engagement, highlighting consistent patterns across genders and editions, with a minor upward trend in problematic use over time.

### 3.3. Net Promoter Score

To assess adolescents’ satisfaction and perceived value of the initiative, participants were asked to rate how likely they would recommend the activity to a friend or peer using the standard Net Promoter Score (NPS) scale (0 = “not at all likely” to 10 = “extremely likely”). Responses (Table 3) were categorized as Promoters (scores 9–10), Passives (scores 7–8), and Detractors (scores 0–6).

Overall, the three-year adolescent sample shows a consistently high level of satisfaction, with NPS values ranging from +69 to +79. Snowball participants tended to report slightly higher NPS than on-site participants, reflecting a positive peer-mediated diffusion effect.

To explore potential links between smartphone engagement (SAS-SV) and perceived value (NPS), we conducted a Chi-square test of independence between SAS-SV risk categories (low involvement, at-risk use, problematic use) and NPS classification (Promoters, Passives, Detractors) across all three years. Results indicated (χ^2^ test, df = 4, *p* < 0.05) indicated a modest but statistically significant association between SAS-SV risk categories and NPS classification. Adolescents with lower or moderate smartphone engagement were more likely to be Promoters, whereas those in the problematic use category showed a slightly higher proportion of Passives and Detractors.

These results demonstrate that the initiative was highly appreciated across adolescent participants, and the socially mediated snowball recruitment likely reinforced positive perceptions. The NPS thus complements SAS-SV findings by highlighting participant satisfaction, perceived relevance, and the potential for peer-driven diffusion.

## 4. Discussion

The discussion is structured to provide a comprehensive interpretation of the findings from the three consecutive editions of the European Researchers’ Night (2023–2025), focusing exclusively on adolescent participants. It begins with a summary of key results and emerging participation patterns (Section 4.1), highlighting the role of on-site and peer-mediated snowball recruitment, the distribution of smartphone engagement levels, and participant satisfaction as measured by the SAS-SV and Net Promoter Score (NPS).

Section 4.2 places these results in the context of prior research, comparing observed patterns of smartphone engagement with international evidence and considering how contextual factors—such as voluntary participation in structured, science-focused events—may modulate risk and protective profiles among adolescents.

Section 4.3 addresses the theoretical and practical implications, exploring how peer networks, structured environments, and CAWI-based feedback mechanisms foster reflective digital practices. Here, both the social diffusion of engagement and the moderating influence of life context and peer/family support are discussed as mechanisms that can promote healthier digital behaviors.

Section 4.4 outlines future directions, identifying opportunities for longitudinal monitoring, targeted interventions for At-Risk adolescents, optimization of peer-mediated strategies, and the integration of mixed-method evaluations to capture richer insights into behavioral trajectories and subjective experiences.

Section 4.5 discusses the limitations of the study, acknowledging constraints related to sample representativeness, potential biases introduced by snowball recruitment, and the specific measurement tools used, while also highlighting how the real-world, stimulating context of the public science event provided unique opportunities for engagement and reflective participation.

Section 4.6 presents practical recommendations for adolescent-centered digital wellbeing initiatives. It emphasizes the potential of integrating CAWI-based surveys into widely used national apps, such as the Italian Io app, to collect timely, anonymized, and representative data on digital behaviors. This approach can enhance youth engagement, provide immediate feedback to foster self-awareness and critical digital literacy, and leverage peer-mediated diffusion to broaden reach. By combining large-scale monitoring with reflective and participatory strategies, these recommendations offer actionable guidance for promoting healthier digital practices among adolescents.

Together, this structure guides the reader through a coherent narrative: from empirical findings to theoretical insights, practical recommendations, and avenues for future research, emphasizing the interplay of context, motivation, and social dynamics in shaping adolescent digital wellbeing.

### 4.1. Summary of Findings and Emerging Patterns

This study focused exclusively on adolescents aged 10–19 years who participated across three consecutive editions of the European Researchers’ Night (2023–2025) in the Rome metropolitan area and surrounding towns. A total of 807 adolescents contributed data, collected both on-site during the events and via peer-mediated snowball recruitment. The analysis of participation patterns revealed that the snowball recruitment mechanism played an increasingly important role over time. On-site participants acted as multipliers, recruiting an average of 2.9 additional peers in 2023 and 4.8 in 2025. Responses continued to arrive for up to three weeks after each event, reflecting an initial rapid engagement followed by a gradual decline. Gender distribution was balanced, and middle school students consistently represented the majority of the sample, highlighting the broad reach of the initiative among early adolescents.

Smartphone engagement, assessed through the SAS-SV, indicated that most adolescents fell into the low involvement or at-risk categories. Problematic use remained a smaller proportion of the sample, though a slight increase over time was observed, particularly among male participants. Chi-square analyses confirmed the stability of these patterns across the three editions and the predominance of low involvement and at-risk use, with minimal gender differences. These results suggest that while most adolescents use smartphones in a non-problematic way, a subset exhibits risk behaviors that require attention.

Evaluation of the initiative through the Net Promoter Score (NPS) revealed consistently high levels of satisfaction across years and participation modes, with snowball participants reporting slightly higher NPS values. A modest but statistically significant association between SAS-SV categories and NPS classifications emerged, indicating that adolescents with lower or moderate smartphone engagement were more likely to act as Promoters, while those with problematic use included a slightly higher proportion of Passives and Detractors. This finding suggests that patterns of smartphone engagement may influence participants’ perceived value of the initiative and their willingness to recommend it to peers.

Taken together, these results highlight three central dynamics. First, peer-mediated recruitment significantly amplifies participation, emphasizing the social diffusion of engagement in adolescent populations. Second, smartphone use patterns are largely stable, with low involvement and at-risk behaviors predominating, though problematic use shows a gradual upward trend. Third, participant satisfaction is high overall, but subtly differentiated according to engagement patterns, suggesting that adolescents’ experiences of digital health initiatives may be linked to their own smartphone behaviors.

This discursive overview provides a coherent framework for interpreting the findings in relation to prior research, examining theoretical and practical implications, and outlining directions for future investigation. It also clarifies that the study population is exclusively adolescents, addressing previous conceptual ambiguities regarding age range.

### 4.2. Key Findings in Relation to Prior Research

In the pooled sample (2023–2025; N = 807), 46.3% of adolescents were classified as Low Involvement, 39.0% as At-Risk Use, and 14.6% as Problematic Use according to the SAS-SV. These findings indicate that, within this science-event-engaged adolescent population and its peer networks, the majority of participants exhibited low to moderate smartphone engagement, while a smaller—though not negligible—proportion met criteria for problematic use.

The distribution remained relatively stable across the three consecutive years, with Problematic Use ranging between 12% and 17% depending on year and gender. This consistency suggests that the observed pattern reflects a structured characteristic of this context rather than a temporary fluctuation. At the same time, the sizeable At-Risk segment (approximately four out of ten adolescents) highlights the presence of an intermediate group that may be particularly relevant for preventive strategies.

When compared with other empirical studies, the proportion of problematic smartphone use observed in the present sample (14.6%) appears lower than figures reported in several international contexts. For example, problematic smartphone use has been reported at 38.1% among Austrian adolescents (Mayerhofer et al., 2024), 55% among South African university students (Mostert, 2025), and 30.1% among southern Italian secondary-school students (Cali et al., 2024). These substantial differences across studies likely reflect variations in age composition, educational level, recruitment strategies, sociocultural environments, and sample characteristics. In particular, the inclusion of university students in Mostert (2025) and the broader school-based sampling frames in Mayerhofer et al. (2024) and Cali et al. (2024) may capture populations with different digital engagement patterns compared to adolescents voluntarily participating in a public science outreach event.

The comparatively lower prevalence observed in the present study may therefore be interpreted within a context-sensitive framework. Participation in structured educational initiatives—and diffusion through socially connected peer networks—may reflect underlying characteristics such as higher academic engagement, interest in learning, or greater reflective awareness of digital habits. Although causal inference cannot be drawn, prior research provides converging evidence that structured lifestyles and psychosocial resources are associated with lower problematic smartphone use. For instance, problematic smartphone engagement measured with the SAS-SV has been shown to be inversely associated with physical activity and positively associated with sedentary behavior and non-study screen time (Jeong et al., 2023). Longitudinal research further indicates that higher levels of psychological capital, including self-control, resilience, and hope, predict lower problematic mobile phone use over time (Ji et al., 2025). Additionally, perceived parental support has been found to reduce problematic use indirectly by enhancing self-esteem and decreasing fear of missing out (FoMO) (Kim, 2022), while network analyses identify self-control and parent–child relationship quality as central variables within problematic smartphone use structures (Huang et al., 2021).

Taken together, these findings support the interpretation that adolescent smartphone engagement is highly heterogeneous and strongly influenced by contextual and relational factors. Rather than contradicting existing literature, the present results complement it by illustrating how sample motivation, voluntary participation, and socially structured engagement may correspond to lower vulnerability profiles. At the same time, the presence of a meaningful At-Risk segment underscores the importance of preventive approaches focused on digital literacy, self-regulation, and balanced use, rather than assuming uniformly high levels of problematic engagement across adolescent populations.

### 4.3. Theoretical and Practical Implications

The present study provides new insights into adolescent smartphone engagement in the context of public science outreach. Across three consecutive editions (2023–2025), the majority of participants displayed Low Involvement (46–48%) or At-Risk Use (37–43%), with a smaller proportion falling in the Problematic Use category (12–17%). These patterns were remarkably stable across years and genders, suggesting a consistent behavioral tendency among youth voluntarily engaging in educational and peer-mediated activities.

From a theoretical perspective, these findings support the view that smartphone engagement is highly context-dependent and heterogeneous (Ferretti et al., 2023; Fischer-Grote et al., 2021; Girela-Serrano et al., 2024; Peiris-John et al., 2020; Santos et al., 2023; Wacks & Weinstein, 2021; Jeong et al., 2023; Ji et al., 2025; Kim, 2022; Huang et al., 2021). Adolescents participating in a voluntary, science-focused initiative may possess higher critical awareness, interest in learning, and reflective attitudes toward their digital habits. Such traits, together with the structured context of participation, likely act as protective factors, moderating the transition from casual or moderate use toward problematic patterns. This aligns with prior evidence showing that self-monitoring, feedback, and awareness reduce the likelihood of risk behaviors (Stewart et al., 2022; Grist et al., 2018; Santos et al., 2023; Wacks & Weinstein, 2021). Moreover, the use of CAWI tools for digital self-assessment enabled participants to track and reflect on their behaviors autonomously, in familiar environments, supporting exploratory engagement and reducing defensive responding (Stewart et al., 2022; Grist et al., 2018).

The peer-mediated dissemination strategy further reinforced these mechanisms. Across editions, adolescents not only participated on-site but actively shared the survey link, producing a snowball effect that extended both the reach and the temporal window of engagement. High satisfaction was consistently observed, with Net Promoter Scores ranging from +69 to +79. Snowball participants reported slightly higher NPS than on-site attendees, reflecting the positive effect of peer-mediated diffusion and the value placed on self-reflection and shared social learning. This upward trend underscores growing engagement and endorsement of the initiative among adolescents over time. Peer networks thus served as vehicles of normalization, enabling reflective practices to circulate naturally among friends, and supporting theoretical models emphasizing the role of social reinforcement and distributed learning in adolescent digital behavior (Dodd et al., 2022; Brinsley et al., 2025; McHale et al., 2022; Llauradó et al., 2021).

From a practical standpoint, these results suggest several implications for the design of digital health initiatives targeting youth. The substantial proportion of at-risk users (~40%) highlights a critical window for preventive interventions aimed at maintaining non-problematic engagement and promoting digital literacy. Embedding feedback mechanisms, self-monitoring tools, and reflective exercises within accessible digital platforms can foster autonomous regulation and improve adolescents’ awareness of their own habits (Stewart et al., 2022; Grist et al., 2018; Santos et al., 2023; Wacks & Weinstein, 2021).

High satisfaction and feedback were measured using a single-item Net Promoter Score (NPS) embedded in the survey (Qualtrics, 2025; SurveyMonkey, 2025; CustomerGauge, 2025).

Additional practical considerations highlight several strategies to support healthy smartphone engagement among adolescents. First, structured life contexts act as protective factors: participation in educational, physical, or socially organized activities can buffer against problematic use and reinforce positive routines. Second, leveraging peer and family support is important, as engagement that involves peers or encourages parental involvement can enhance self-esteem, reduce FoMO, and strengthen preventive effects (Kim, 2022; Huang et al., 2021). Finally, monitoring and iterative adaptation of interventions is essential: tracking SAS-SV categories and NPSs provides actionable feedback for refining programs. Notably, the increase in NPS across editions indicates that adolescents not only participate actively but also perceive value in these initiatives.

Overall, the combination of voluntary engagement, peer-mediated diffusion, structured contexts, and embedded feedback constitutes a coherent, replicable framework for adolescent digital wellbeing promotion. This approach shifts the focus from a pathology-centered perspective toward reflective autonomy, highlighting how context, motivation, and social networks can modulate risk and support healthier smartphone habits.

### 4.4. Future Directions

The findings of this study open several promising avenues for future research and intervention design in adolescent digital wellbeing. First, the observed stability of smartphone engagement patterns across three consecutive years suggests the value of longitudinal monitoring. Following cohorts over time would allow researchers to examine not only the evolution of digital behaviors but also the long-term effects of participation in structured educational initiatives. Previous research has highlighted that adolescents’ reflective engagement with digital health tools can be sustained when monitored longitudinally, providing a clearer picture of behavioral trajectories (Kwon et al., 2013; Stewart et al., 2022; Grist et al., 2018).

Second, the peer-mediated dissemination mechanism demonstrated in this initiative proved highly effective in both expanding reach and reinforcing reflective practices. Future studies could explore variations in peer-led strategies, including different messaging formats, incentives, and interactive feedback, to maximize the adoption and normalization of healthy digital behaviors. Evidence from peer-led health interventions indicates that adolescents are more likely to internalize reflective habits when encouraged within trusted social networks rather than through formal institutional channels (Dodd et al., 2022; Brinsley et al., 2025; McHale et al., 2022; Llauradó et al., 2021).

Third, the sizable proportion of participants classified as At-Risk underscores the importance of targeted preventive strategies. Interventions could integrate self-monitoring tools, digital wellbeing education, and awareness campaigns that specifically address this intermediate group, aiming to prevent escalation toward problematic use. This approach aligns with recent evidence showing that early engagement with digital self-regulation practices can buffer against the development of maladaptive smartphone behaviors (Giansanti, 2025; Jeong et al., 2023; Ji et al., 2025).

Finally, future initiatives would benefit from a mixed-method evaluation framework. Combining quantitative measures, such as the SAS-SV and NPS, with qualitative insights from participant reflections would provide a richer understanding of subjective experiences, peer influence, and perceived value of the intervention. Research suggests that when adolescents can track and reflect on their own behaviors in familiar, non-evaluative contexts, they report more authentic and actionable insights, which can guide personalized intervention design (Stewart et al., 2022; Grist et al., 2018; Llauradó et al., 2021).

Taken together, these directions emphasize a multi-layered approach: leveraging longitudinal observation, optimizing peer-mediated engagement, focusing on at-risk youth, and integrating mixed-method evaluation. Such a strategy not only enhances scientific understanding but also strengthens the practical impact of digital wellbeing initiatives, supporting adolescents in cultivating reflective, balanced, and socially functional smartphone habits.

### 4.5. Limitations

Despite the strengths of the initiative, several contextual and methodological considerations should be acknowledged. First, the study was conducted within a public science event in Rome, attracting adolescents and young adults who were already motivated to engage with science-related activities. This context may limit the generalizability of the findings to the broader national or international youth population. At the same time, the event setting provided a stimulating and supportive environment, offering opportunities for curiosity-driven participation, peer interaction, and reflective engagement, which likely enhanced both recruitment and the quality of responses (Stewart et al., 2022; Grist et al., 2018; Bond, 2013).

Second, the snowball diffusion mechanism extended participation beyond the physical event, increasing reach and enabling ongoing engagement. However, it may have introduced network and self-selection biases, as adolescents who chose to participate and share the survey could differ systematically from non-participants in terms of motivation, interest in science, or digital habits. Nevertheless, this peer-mediated strategy also represents a notable strength: it amplified engagement, fostered reflective practices, and demonstrates how non-stigmatizing initiatives can propagate organically through adolescent networks (Dodd et al., 2022; Brinsley et al., 2025; McHale et al., 2022; Llauradó et al., 2021).

Finally, regarding measurement, the Smartphone Addiction Scale–Short Version (SAS-SV) was used to classify participants into Low Involvement, At-Risk, and Problematic Use categories (Kwon et al., 2013). While alternative validated instruments exist for assessing problematic smartphone and internet-related behaviors—such as the Smartphone Application-Based Addiction Scale (SABAS), the Bergen Social Media Addiction Scale (BSMAS), and other brief digital-use measures (Chen et al., 2020; Tung et al., 2022)—the computer-assisted web interview (CAWI) format offered high flexibility, allowing validated tools to be integrated according to specific research objectives and contexts. Its self-administered, mobile-friendly, and anonymous design facilitated honest responses and can be adapted in future implementations to suit different populations while maintaining participant comfort and engagement (Sutter & Perrin, 2007).

Taken together, these considerations highlight the balance between methodological limitations and the contextual advantages of conducting research in a real-world, engaging, and socially dynamic setting. Future studies should aim to replicate these findings in more diverse populations while preserving the stimulating, reflective environment that characterizes the initiative.

### 4.6. Recommendations for Adolescent-Centered Digital Wellbeing Initiatives

The present initiative suggests that CAWI-based assessments are highly suitable for youth-centered digital wellbeing interventions. Building on this, national apps already in widespread use—such as the Italian *Io* app—could provide an effective infrastructure for large-scale, mobile-friendly data collection on digital behaviors.

During the COVID-19 pandemic, apps like *Io* demonstrated the potential of mobile platforms to reach millions of users quickly for health-related purposes. However, at present, these apps are underutilized for monitoring digital habits or promoting reflective engagement on smartphone use, particularly among adolescents and young adults. Integrating CAWI-style surveys into existing national apps could allow authorities to collect timely, anonymized, and representative data on digital behaviors, extending beyond isolated research events.

Such integration would have multiple advantages:

- Youth engagement: Young people are already familiar and comfortable with app-based interactions, increasing participation rates and the authenticity of responses.
- Scalability: National apps reach large populations at low cost, allowing continuous or periodic monitoring of digital habits.
- Immediate feedback and reflection: Embedded surveys could provide real-time, personalized feedback, transforming data collection into an experiential intervention that promotes self-awareness and critical digital literacy.
- Peer-mediated diffusion: Features encouraging social sharing or reflection could leverage network effects, further expanding reach, as observed in the European Researchers’ Night snowball effect.

In summary, leveraging national apps like *Io* for CAWI-based surveys could complement existing public health and educational strategies, turning widely used digital infrastructures into platforms for monitoring, awareness-building, and promoting reflective digital citizenship among youth. This approach offers a promising pathway to scale evidence-based interventions while engaging young people as active participants in understanding and managing their own digital behaviors.

## 5. Conclusions

This study examined adolescent smartphone engagement across three consecutive editions (2023–2025) of the European Researchers’ Night in Rome, involving 807 participants recruited on-site and via peer-mediated snowball diffusion. Findings indicate that most adolescents exhibited low or at-risk smartphone use, with a smaller proportion showing problematic patterns. Participation was amplified by peer networks, with snowball recruitment extending both reach and engagement duration. High satisfaction levels, reflected in consistently strong Net Promoter Scores (+69 to +79), suggest that adolescents valued the opportunity for self-reflection and shared social learning.

The study contributes to understanding adolescent digital behavior by highlighting the interplay of structured educational contexts, peer influence, and self-monitoring in promoting reflective and balanced smartphone use. Methodologically, the initiative demonstrates the feasibility and effectiveness of CAWI-based assessments for youth-centered digital wellbeing interventions.

Future research should explore longitudinal monitoring, targeted interventions for at-risk youth, and the integration of mixed-method evaluation to capture subjective experiences and peer dynamics. Leveraging these insights can inform scalable, evidence-based strategies that foster digital literacy, reflective autonomy, and healthier smartphone habits among adolescents.

## Figures and Tables

**Table 1:** Participant distribution by year, mode of participation, and demographics.

Year	On-Site	Snowball	Total	Age Mean ± SD	Female %	Male %	School Level (M/H)	Snowball Effect
2023	48	139	187	16.5 ± 1.9	54	46	M: 78%, H: 22%	2.90
2024	59	201	260	16.7 ± 2.0	52	48	M: 75%, H: 25%	3.40
2025	62	298	360	16.9 ± 2.1	53	47	M: 74%, H: 26%	4.80

**Table 2:** SAS-SV Risk Classification for Adolescent Participants by Year and Gender (On-site + Snowball).

Year	Gender	N	Low Involvement	At-Risk Use	Problematic Use
2023	Female	101	47 (46%)	41 (41%)	13 (13%)
2023	Male	86	38 (44%)	36 (42%)	12 (14%)
2024	Female	135	63 (47%)	51 (38%)	21 (15%)
2024	Male	125	56 (45%)	54 (43%)	15 (12%)
2025	Female	191	92 (48%)	71 (37%)	28 (15%)
2025	Male	169	78 (46%)	62 (37%)	29 (17%)

**Table 3:** NPSs for Adolescent Participants by Year and Participation Mode.

Year	Mode	N	Promoters	Passives	Detractors	NPS
2023	On-site	48	36 (75%)	9 (19%)	3 (6%)	+69
2023	Snowball	139	108 (78%)	25 (18%)	6 (4%)	+74
2024	On-site	59	44 (75%)	12 (20%)	3 (5%)	+70
2024	Snowball	201	162 (81%)	35 (17%)	4 (2%)	+79
2025	On-site	62	48 (77%)	12 (19%)	2 (4%)	+73
2025	Snowball	298	240 (81%)	50 (17%)	8 (3%)	+78

## Data Availability

All data supporting the findings of this study are included within the manuscript.
